# Histopathological Characteristics of Placenta in Pregnancies Complicated by Intrauterine Growth Restriction—A Pilot Study

**DOI:** 10.3390/diagnostics16010060

**Published:** 2025-12-24

**Authors:** Liviu Moraru, Raluca Moraru, Diana Maria Chiorean, Septimiu Voidăzan, Lorena Solovăstru, Melinda-Ildiko Mitranovici

**Affiliations:** 1 Department of Anatomy, “George Emil Palade” University of Medicine, Pharmacy, Sciences and Technology, 540142 Targu Mures, Romania; liviu.moraru@umfst.ro (L.M.); raluca.moraru@umfst.ro (R.M.); 2 Department of Pathophysiology, “George Emil Palade” University of Medicine, Pharmacy, Science, and Technology of Targu Mures, 38 Gheorghe Marinescu Street, 540142 Targu Mures, Romania; 3 Department of Pathology, County Clinical Hospital of Targu Mures, 540072 Targu Mures, Romania; 4 Department of Epidemiology, “George Emil Palade” University of Medicine, Pharmacy, Sciences and Technology, 540142 Targu Mures, Romania; septimiu.voidazan@umfst.ro; 5 Normal and Pathological Morphology Laboratory, Center for Advanced Medical and Pharmaceutical Research, “George Emil Palade” University of Medicine, Pharmacy, Sciences and Technology, 540142 Targu Mures, Romania; lorenasolovastru3@gmail.com; 6 Doctoral School of Medicine and Pharmacy, “George Emil Palade” University of Medicine, Pharmacy, Science, and Technology of Targu Mures, 540142 Targu Mures, Romania; mitranovicimelinda@yahoo.ro; 7 Department of Obstetrics and Gynecology, Emergency County Hospital Hunedoara, 14 Victoriei Street, 331057 Hunedoara, Romania

**Keywords:** IUGR, Doppler, placental pathology, immunohistochemistry

## Abstract

**Background/Objectives:** Intrauterine growth restriction (IUGR) is a condition in which a fetus does not reach its normal growth potential and is associated with increased neonatal morbidity. Surveillance relies on cardiotocography, a biophysical ultrasound, and a Doppler assessment, but placental pathology remains insufficiently integrated into clinical evaluations. This study aimed to compare placentas from IUGR and normal pregnancies. **Methods:** This cohort included 34 pregnancies (16 IUGR, 18 controls) managed at Hunedoara County Hospital (Romania). The ultrasound and Doppler parameters were documented. The placentas were collected after delivery, fixed in formalin, and processed using standard histopathological protocols. The villous morphology and maternal vascular malperfusion features were assessed on H&E sections, focusing on syncytial knots, villous caliber reduction, stromal fibrosis, fibrin deposition, and infarctions. Immunohistochemistry for CD34, cytokeratin 7 (CK7), CD68, vascular endothelial growth factor (VEGF), and Hypoxian inducible factor 1 (HIF-1α)was performed using a semi-quantitative 0–3 scoring system. A statistical analysis was performed using chi-squared testing for categorical variables and t-tests for continuous variables. **Results:** The ultrasound evaluation showed an estimated fetal weight below the 10th percentile and abnormal Doppler indices in the IUGR group. The histopathology demonstrated a strong association between IUGR and villous abnormalities, including an increased number of syncytial knots, stromal fibrosis, a reduced villous caliber, and placental infarctions. The immunohistochemistry showed a marked overexpression of VEGF and HIF-1α and increased CD68-positive Hofbauer cells in IUGR placentas (*p* < 0.0001), while CD34 and CK7 displayed preserved strong staining in both groups. **Conclusions:** Placentas from IUGR pregnancies exhibited advanced maternal vascular malperfusion with consistent hypoxic and inflammatory changes, correlating with Doppler alterations. These findings highlight the diagnostic relevance of placental pathology in pregnancies with IUGR.

## 1. Introduction

Intrauterine growth restriction (IUGR) is a condition in which a fetus does not reach its normal growth potential [1,2,3]. IUGR is a common pregnancy complication with high neonatal morbidity and mortality [2]. Its prevalence is 7–15% worldwide [3]. Early-onset preeclampsia (PE) is the most important cause of IUGR [1]. The associated risks include placental abruption, eclampsia, and multisystem organ failure or, in the long term, develop cardiovascular disease [1]. In addition, the offspring of women who had early-onset PE associated with IUGR are at an increased risk for cardiovascular (CV) disease later in life [4]. In many cases, maternal or fetal complications are present at the time of diagnosis due to an advanced disease state [5].

The exact etiology of IUGR is unknown. Moreover, there are no effective interventions to treat PE or alleviate IUGR. Placental impairment and endothelial dysfunction with the inhibition of angiogenesis and impaired spiral artery remodeling leading to placental ischemia are critical to fetal growth and development. This is associated with a variety of adverse perinatal outcomes [4,5,6,7]. The ischemic placenta releases pro-inflammatory mediators and hypoxia-driven signaling molecules that contribute to widespread maternal endothelial dysfunction, supporting the concept that PE with IUGR represents a multisystem disorder [8].

There is a lack of consensus between international guidelines, the Society for Maternal–Fetal Medicine (SMFM), and the International Society of Ultrasound in Obstetrics and Gynecology (ISUOG) regarding the definition, etiology, and diagnostic criteria for fetal growth restriction [9,10]. The SMFM describes IUGR as an estimated fetal weight (EFW) or abdominal circumference (AC) < 10th percentile, while the ISUOG includes either an EFW or AC < 3rd percentile or an EFW or AC < 10th percentile combined with abnormal Doppler findings or a decrease in the growth centiles [9]. The ISUOG endorses the Delphi criteria and combines size thresholds with Doppler velocimetry to differentiate IUGR from small-for-gestational-age fetuses for being at risk of adverse outcomes [2]. This uncertainty leads to questionable management regarding the prediction of neonatal IUGR, and also for the optimal timing of delivery for a fetus with IUGR [9,10]. The neonatal complications include respiratory distress, intraventricular hemorrhage, a cord blood pH < 7.1, seizures, admission to the neonatal intensive care unit, or neonatal death [9]. Accurate surveillance followed by delivery at an appropriate gestational age is critical for improving outcomes. Cardiotocography (cCTG), an ultrasound biophysical profile, and Doppler velocimetry are the most important tools for monitoring a fetus with IUGR to reduce the chance of a poor neonatal outcome [11]. However, the identification of potential biomarkers may help with the early detection of this pathology, improving the prognosis [5]. Placental findings are mandatory in the process of understanding the molecular mechanisms of this disease and are also necessary for identifying future biomarkers and therapies [1,12,13].

The aim of this study was to evaluate the differences between the placentas of pregnancies with IUGR and those of normal pregnancies. Biophysical measurements and Doppler velocimetry were used to identify IUGR. The placental architecture and villous microcirculation were assessed, along with the expression of hypoxia-related (HIF-1α), angiogenesis-related (VEGF), trophoblastic (CK7), endothelial (CD34), and macrophage (CD68) markers.

## 2. Materials and Methods

IUGR was examined in pregnant women referred to County Hospital Hunedoara (Romania) in this cohort study. The control group included women with normal pregnancies. IUGR was defined according to established clinical and ultrasonographic criteria, including an estimated fetal weight or birth weight below the 10th percentile, supported by a Doppler evaluation when available.

This study received approval from the Ethics Committee of the Center for Advanced Medical and Pharmaceutical Research of UMFST “George Emil Palade” Târgu Mureș (No. 3761/30 April 2025). All the procedures complied with the Declaration of Helsinki and national regulations. Written informed consent was obtained from all the participants. All the patients included in the analysis delivered by cesarean section.

The inclusion criteria for the study group were singleton pregnancies that fulfilled the diagnostic criteria for IUGR according to the ISUOG definition (estimated fetal weight < 10th centile based on sonographic measurements, and Doppler velocimetry), the availability of complete clinical and ultrasonographic documentation, and signed informed consent. The inclusion criteria for the control group were pregnant women who agreed to participate in the study regardless of their risk factors for IUGR, allowing comparisons of the study group with pregnancies in general. The exclusion criteria included non-IUGR pregnancies, multiple gestations, the absence of required ultrasound evaluations, known fetal structural or chromosomal abnormalities, intrauterine infections, immediate delivery for unrelated clinical emergencies, natural delivery, and loss to follow-up.

A total of 34 patients were included in the final analysis: 16 in the IUGR group and 18 in the control group.

Maternal data (age, education, environment, gravidity, parity, gestational age, BMI, smoking habits, and known comorbidities such as diabetes, chronic hypertension, thrombophilia, or hypothyroidism) were retrieved from medical records. The gestational age was determined according to first-trimester crown–rump length measurements.

The neonatal outcomes included Apgar scores, birth weight, sex, and NICU admission. Additional early neonatal complications that were documented were respiratory distress, the need for mechanical ventilation, infections, intraventricular hemorrhage, seizures, a phototherapy requirement, blood transfusions, hypoxic–ischemic encephalopathy, and neonatal death.

### 2.1. IUGR and Ultrasound Protocols

Ultrasound examinations were performed and documented by a maternal–fetal medicine expert using a Voluson S10 Expert sonography machine (GE Healthcare, Milwaukee, WI, USA) via a transabdominal ultrasound. The fetal ultrasonographic assessment included biophysical measurements and Doppler parameters such as the uterine artery pulsatility index (PI), second trimester and notch, and the middle cerebral artery resistivity index (RI). All the measurements were conducted in accordance with the protocols established by the International Society of Ultrasound in Obstetrics and Gynecology (ISUOG). The estimated fetal weight percentiles were calculated according to Intergrowth-21st [10]. The uterine artery PI and cerebral artery RI were examined using Doppler velocimetry and calculated according to a standard protocol of the current international Doppler guidelines. The uterine artery PI was considered abnormal when it was > 95th percentile. A notch sign of the uterine artery was recorded. The cerebral artery resistivity index was set with a cut-off of 0.54; values less than this number were predictive of IUGR [10].

### 2.2. Placenta Examination

Placental specimens were collected immediately after delivery. The placental weight was recorded, and all the tissues were fixed in 10% neutral-buffered formalin for a minimum of 24 h before processing. Gross and microscopic evaluations were performed at the Center for Advanced Medical and Pharmaceutical Research (CCAMF), part of the George Emil Palade University of Medicine, Pharmacy, Science and Technology of Târgu Mureș.

The placental examination followed the institutional protocol in accordance with the Amsterdam Placental Workshop Group Consensus Statement [14]. Sampling included the umbilical cord, the fetal membranes, and up to six full-thickness sections of the placental disc, incorporating both maternal and fetal surfaces. After fixation, the tissues were routinely processed, embedded in paraffin, sectioned at 3–4 μm, and stained with hematoxylin and eosin (H&E).

The histopathological assessment focused on the villous morphology, the stromal composition, and vascular changes, with particular attention to lesions associated with maternal vascular malperfusion. The features evaluated included the degree of syncytial knot formation, the presence of chorangiosis, intravillous calcifications, and both early and extensive placental infarctions.

No formal semi-quantitative scoring system was applied to the H&E sections; instead, the lesions were described qualitatively based on their presence, distribution, and morphological severity.

### 2.3. Immunohistochemical Assessment

An immunohistochemical evaluation was performed on 3–4 μm FFPE sections mounted on positively charged slides. After deparaffinization and rehydration, endogenous peroxidase activity was blocked using hydrogen peroxide. Heat-induced epitope retrieval was carried out with either citrate- or EDTA-based Leica BOND retrieval buffers, selected according to antibody-specific recommendations.

Primary antibodies targeting CD34, CK7, CD68, VEGF, and HIF-1α were purchased from Abcam (Cambridge, UK) and applied using manufacturer-recommended dilutions and incubation times. Staining was performed on a fully automated Leica BOND immunostainer using the BOND Polymer Refine Detection system (DAB). Chromogenic visualization was achieved with 3,3′-diaminobenzidine (DAB), followed by counterstaining with Mayer’s hematoxylin.

Endogenous tissue elements served as internal positive controls (endothelium for CD34, trophoblast for CK7, stromal macrophages for CD68, and syncytiotrophoblast for VEGF and HIF-1α). Negative controls were processed by omitting the primary antibody. After staining, the slides were examined using a Zeiss optical microscope (Zeiss, Jena, Germany) at 4×, 10×, 20×, and 40× objectives.

All immunohistochemical markers were evaluated using a standardized semi-quantitative 0–3 intensity scoring system, where 0 indicates the absence of staining, 1 indicates weak or focal staining, 2 indicates a moderate intensity, and 3 indicates strong, diffuse positivity. The full 0–3 semi-quantitative scoring system was applied; however, within this cohort, the staining patterns occurred exclusively at the extremes of the scale. No markers demonstrated an intermediate (score 2) intensity, and therefore, only scores 0, 1, and 3 were recorded in the final dataset. This distribution reflects the polarized nature of the placental tissue response in advanced maternal vascular malperfusion.

The CD34 expression was assessed based on the endothelial staining intensity and its distribution in the most vascularized villous regions. The CK7 scoring reflected the cytoplasmic intensity and continuity of trophoblastic staining. The CD68 scoring was based on the Hofbauer cell density within the villous stroma, evaluated across multiple high-power fields. The VEGF and HIF-1α expression were assessed based on the cytoplasmic staining intensity within the syncytiotrophoblast and villous stromal cells.

All the immunohistochemical slides were independently evaluated by two observers blinded to the clinical data, with discordant cases reviewed at a multi-headed microscope until a consensus was reached.

### 2.4. Statistical Analysis

A statistical analysis was performed using the Statistical Package for Social Sciences (SPSS, version 23, Chicago, IL, USA). The data were labelled as nominal or quantitative variables. Nominal variables were characterized by means of frequencies. Quantitative variables were tested for the normality of their distribution using the Kolmogorov–Smirnov test and were described by the mean ± standard deviation. The frequencies of nominal variables were compared with a chi-squared test. Differences in the means between groups were analyzed using a t-test. The level of statistical significance was set at *p* < 0.05.

## 3. Results

The demographic variables did not differ significantly between the IUGR and control groups. The Doppler assessment demonstrated marked hemodynamic alterations in the IUGR group, including an elevated uterine artery pulsatility index (>95th percentile) and the persistence of early diastolic notching, a pattern typically associated with the subsequent development of preeclampsia. The middle cerebral artery resistivity index values were also significantly reduced (RI < 0.54). Ultrasonography identified an estimated fetal weight below the 10th percentile in all IUGR cases, whereas this finding was rare among the controls. These differences reached statistical significance when using the chi-squared test (Table 1).

In contrast, maternal comorbidities were exclusively observed in the IUGR group, including type II diabetes, gestational diabetes, hypothyroidism, hypertension, and thrombophilia (*p* = 0.007). The APGAR scores and NICU admission rates showed no significant differences between the groups. Apart from premature birth and hypertension, we did not encounter other obstetrical complications such as placental abruption or severe hemorrhage.

Regarding the pathological placental changes encountered, the histopathological evaluation using H&E staining revealed a strong association between IUGR and villous structural abnormalities. A villous caliber reduction, stromal fibrosis, edema, and vascular congestion were present in the IUGR group in a statistically significant proportion.

The degree of syncytial knot formation and the presence of intravillous calcifications were increased in IUGR placentas, but these features do not indicate an infarction itself; rather, they reflect accelerated villous maturation and chronic hypoxic stress.

Early and extensive placental infarctions were independently identified as ischemic lesions and were significantly more frequent in the IUGR group (75% vs. 5.6%; *p* < 0.0001).

Mild abnormalities were exclusive to IUGR placentas, while a normal villous architecture characterized almost all of the controls (94.4%). Excessive perivillous fibrin deposition, intervillous thrombi, and prominent syncytial knot proliferation were significantly more common in IUGR placentas (*p* < 0.0001).

Placental macrophages mainly consist of macrophages distributed among fetal chorionic villi. Two macrophage phenotypes are described in the literature—pro-inflammatory (M1) and anti-inflammatory (M2)—but routine CD68 immunostaining identifies Hofbauer cells without distinguishing the functional subtypes.

In our study, CD68 highlighted an increased Hofbauer cell density in the IUGR group, ranging from mild infiltration (score 1) to extensive accumulation (score 3), whereas the control placentas showed only physiological low-density patterns (score 0) (*p* < 0.0001). No conclusions regarding macrophage polarization or angiogenic impairment can be drawn solely from the CD68 staining.

The IHC investigations showed immunoreactivity for anti-hypoxia-inducible factor (HIF) and anti-vascular endothelial growth factor (VEGF) antibodies in the villous trophoblast and stromal cells of the IUGR placentas.

All the IUGR placentas demonstrated strong, diffuse staining for VEGF (score 3), whereas the controls showed only weak physiological expression (score 1) (*p* < 0.0001).

HIF-1α showed a similar distribution, with strong positivity in the IUGR placentas and absent/weak staining in the controls (*p* < 0.0001), consistent with hypoxia-related upregulation.

CD34 staining demonstrated preserved endothelial reactivity in both groups, without significant intergroup differences. Therefore, it does not indicate an altered microvascular density in this cohort.

Similarly, CK7 showed uniform strong staining of the trophoblastic layer in both groups, with no evidence of trophoblast loss or discontinuity (Table 2).

A continuous variable analysis using an independent samples t-test for the two groups identified statistically significant differences for weight (*p*-value—0.022), BMI (*p*-value—0.0001), weight gain (*p*-value—0.0001), gestational age at delivery (*p*-value—0.0001), fetal birth weight (*p*-value—0.0001), IR MCA (*p*-value—0.0001), and placental weight (*p*-value—0.0001) (Table 3). A statistically significant correlation was therefore found between weight, weight gain and BMI as risk factors for PE and the occurrence of IUGR. Also, the risk of premature birth was increased in the IUGR group and was significantly associated with Doppler changes and placental weight.

The histopathological analysis demonstrated multiple placental lesions characteristic of maternal vascular malperfusion, including enhanced villous maturation with marked syncytial knotting, stromal fibrosis, a villous caliber reduction, areas of infarction, and focal intravillous calcifications. Occasional perivillous fibrin deposition and intervillous thrombi were also identified. The immunohistochemical evaluation showed the strong cytoplasmic expression of HIF-1α and VEGF in IUGR placentas, consistent with a hypoxia-driven response, as well as an increased CD68-positive Hofbauer cell density within the affected villi. These findings support the presence of pronounced malperfusion-related hypoxic and inflammatory changes. The extent of these lesions was correlated with the antenatal Doppler abnormalities observed in the IUGR group.

**Figure 1:**
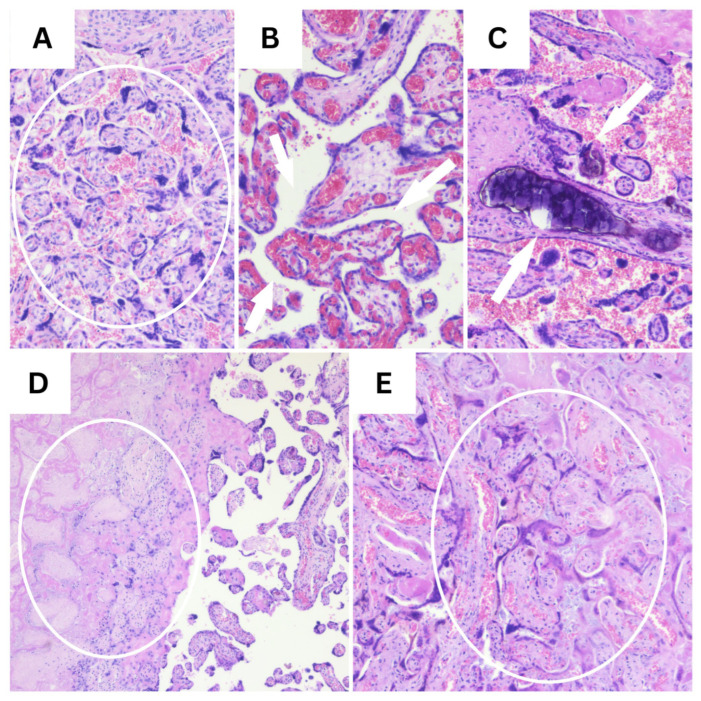
Representative H&E findings in placental tissue (10×). (**A**) Marked syncytial knotting, indicative of accelerated villous maturation (circle). (**B**) Chorangiosis, defined by an increased number of capillaries within terminal villi (white arrows). (**C**) Intravillous calcifications (white arrows). (**D**) Extensive placental infarction showing villous necrosis and the loss of normal architecture (circle). (**E**) Early placental infarction with villous ischemic changes and stromal collapse (circle).

**Figure 2:**
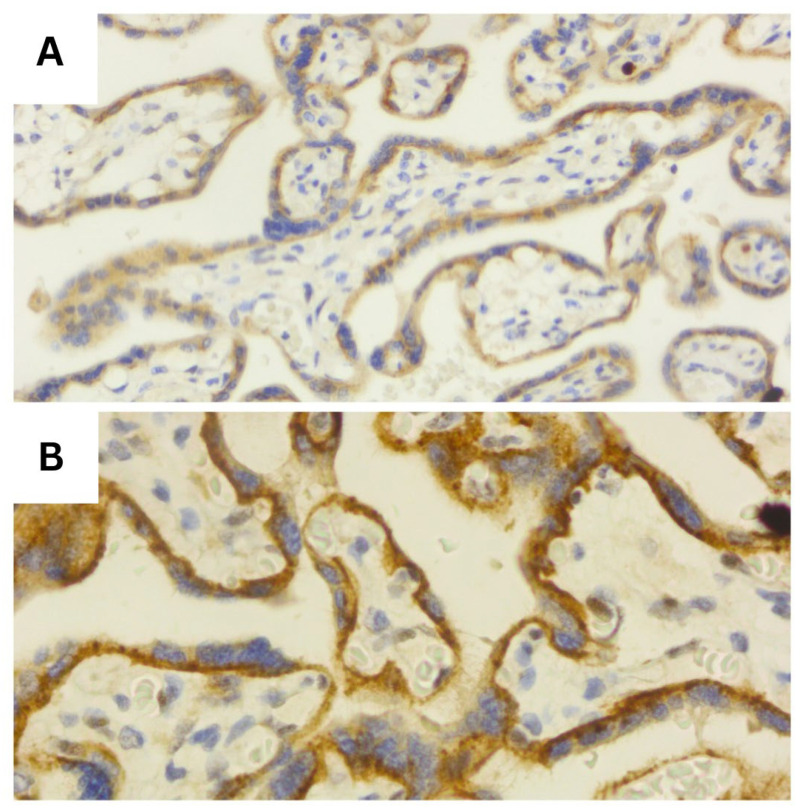
HIF-1α expression illustrates the contrast between (**A**) weak, focal cytoplasmic staining in villous trophoblast (score 1) and (**B**) strong, diffuse cytoplasmic expression (score 3), reflecting accentuated hypoxic signaling in pathological placentas (40×).

**Figure 3:**
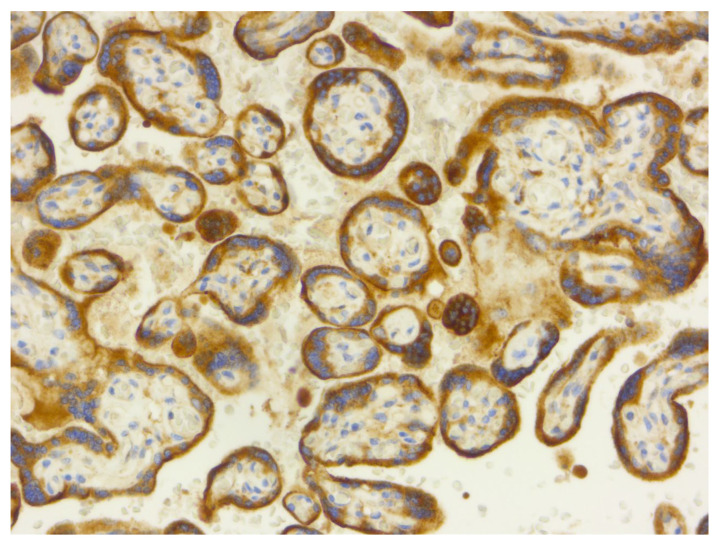
VEGF immunostaining (20×). VEGF immunoreactivity showing strong, diffuse cytoplasmic expression within syncytiotrophoblast and stromal cells (score 3), consistent with marked upregulation in placental hypoxia.

**Figure 4:**
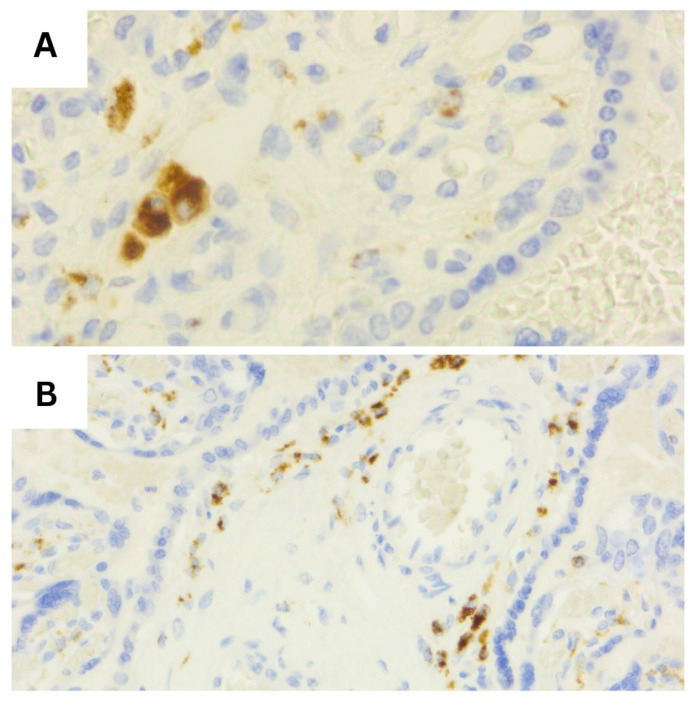
CD68 immunostaining, demonstrating sparse, low-density Hofbauer cells within the villous stroma (score 1, (**A**)) compared with marked macrophage accumulation forming multiple confluent positive aggregates (score 3, (**B**)) (40×).

**Figure 5:**
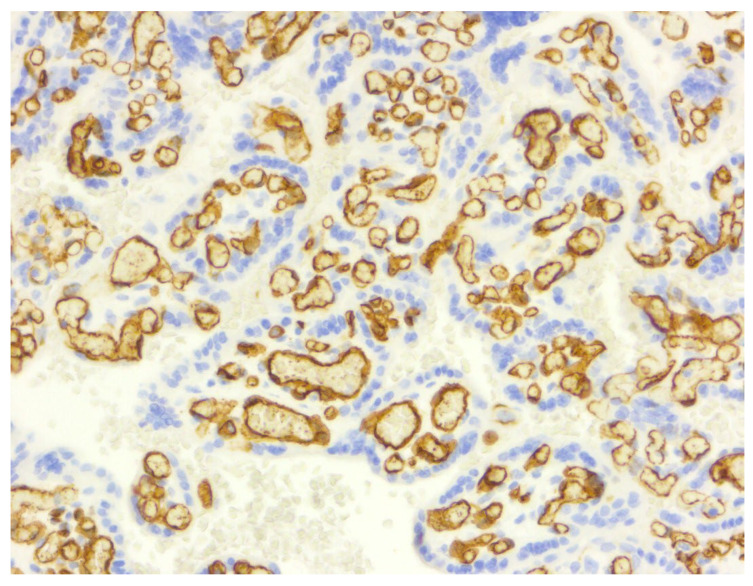
CD34 immunostaining (10×), highlighting a dense network of villous capillaries with strong endothelial staining (score 3), a pattern uniformly preserved across both normal and pathological placentas. This reflects a maintained microvascular architecture.

**Figure 6:**
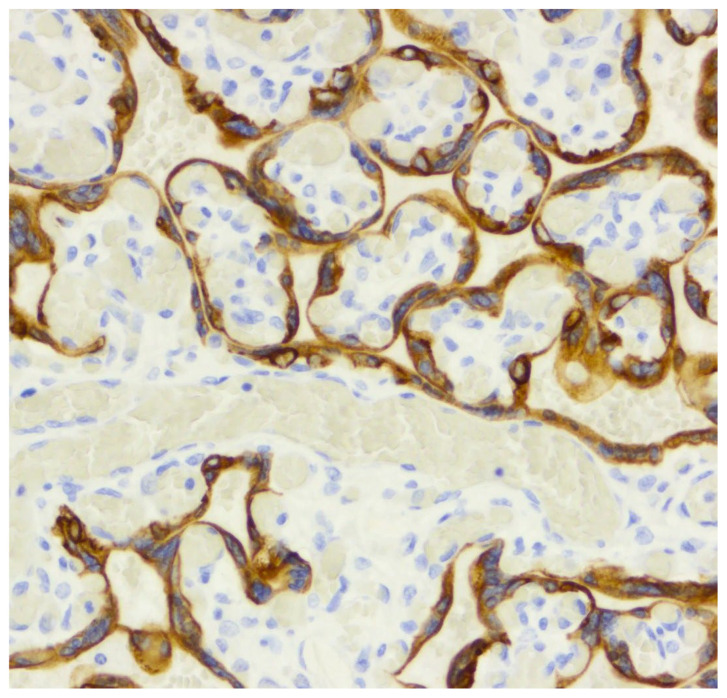
CK7 immunostaining (20×), demonstrating strong, continuous cytoplasmic expression within the villous trophoblast (score 3), a staining pattern consistently present in both normal and IUGR placentas. This indicates a preserved trophoblastic integrity.

Representative histopathological and immunohistochemical images are provided in the figures below (Figure 1, Figure 2, Figure 3, Figure 4, Figure 5 and Figure 6).

## 4. Discussion

Preeclampsia associated with IUGR is a pregnancy disorder that threatens the life and health of the mother and the child [15]. The pathophysiology of IUGR includes maternal, fetal, and placental factors. Placental dysfunction is considered to be the most important cause of severe IUGR. A placental impairment such as ischemia or thrombotic vasculopathy is a common finding in early-onset PE/IUGR [1,12,13]. Our research identified parameters that markedly changed in the IUGR group, such as BMI (*p*-value—0.0001), weight gain (*p*-value—0.0001), gestational age at delivery (*p*-value—0.0001), fetal birth weight (*p*-value—0.0001), IR MCA (*p*-value—0.0001), and placental weight (*p*-value—0.0001). This was correlated with the severity of placental insufficiency observed histologically and immunohistochemically. The crucial role of infarctions and malperfusion in IUGR was also observed by Bujorescu in his research [16].

The definitions of IUGR according to the ISUOG and the SMFM do not make any associations between placental insufficiency and a composite adverse neonatal outcome [17]. Research has been focused on the diagnosis and monitoring of IUGR, without taking into account the pathophysiology of this disease, which may reflect the individual outcomes [17]. Doppler studies have revealed an abnormal flow leading to fetal growth restriction [5]. This abnormal blood flow is due to placental vascular malperfusion (MVM). Placental histopathological findings have shown placental insufficiency because of MVM [17]. This could lead to adverse perinatal outcomes such as respiratory distress syndrome; neonatal intensive care admission; hypoglycemia; intrapartum hypoxia, which requires immediate delivery; stillbirth; or perinatal death [17]. According to our findings, no significant newborn morbidities were described, and no differences were found in the Apgar scores or NICU admission between the IUGR and control groups. The fetal birth weight was significantly lower instead (*p*-value—0.0001). Even if both the SMFM and ISUOG/Delphi criteria had strong screening potential for the detection of infants with a BW <  10th or <3rd percentile, these criteria do not predict adverse neonatal outcomes [18]. The placental histology, along with the released biomarkers, may help in this direction.

Pregnancy and the neonatal outcomes were evaluated in Mansour Ghanaei’s study in two groups: group I comprised cases of PE-induced IUGR (PE-IUGR), and group II comprised cases of idiopathic IUGR (I-IUGR). Low birth weight and preterm delivery were higher in the PE-IUGR group (*p* < 0.001 in all) than in the I-IUGR group. Placental malperfusion was also higher in the PE-IUGR group (*p* > 0.001) than in the I-IUGR group, leading to the conclusion that placental ischemia with reduced blood flow to the fetus might lead to low birth weight and preterm delivery [1].

According to Liu’s study, diminished HIF-1α expression during the placental ischemic process leads to excessive apoptosis, the reduced expression of VEGF and PlGF, and the overexpression of sFlt1, sEng, and TNF-α, leading to endothelial dysfunction and elevated systolic blood pressure [7]. Placental histology in PE showed the reduced capillarization of the terminal villi and increased apoptosis and necrosis in cases of IUGR [19]. Angiogenic factors, such as vascular endothelial growth factor A (VEGFA), placental growth factor (PlGF) [19,20], and hypoxia-inducible factor 1 subunit alpha (HIF1A) [21], are among the factors reported to be involved in vessel formation in terminal villi [19]. The symptoms regress after the delivery of the placenta, pointing out its central role [22].

In our research, a histopathological evaluation using H&E staining revealed classic features of maternal vascular malperfusion rather than “villitis,” including a villous caliber reduction, stromal fibrosis, increased syncytial knotting, fibrin deposition, and early or extensive infarctions. Severe lesions consisting of stromal fibrosis, a villous caliber reduction, and features of advanced maternal vascular malperfusion were present in 75% of the IUGR placentas, but in only one control sample (5.6%; *p* < 0.0001). Mild abnormalities were exclusive to the IUGR placentas, while a normal villous architecture characterized almost all of the controls (94.4%). Excessive perivillous fibrin deposition, intervillous thrombi, and marked syncytial knot proliferation were significantly more common in IUGR placentas (*p* < 0.0001).

The immunohistochemical findings are presented in Table 2. The VEGF expression differed markedly between groups: all of the control placentas exhibited weak physiological reactivity (score 1), whereas all of the IUGR placentas demonstrated strong, diffuse staining (score 3) (*p* < 0.0001). Importantly, this pattern reflects reactive upregulation in the syncytiotrophoblast under hypoxic stress, not “anti-angiogenic activity”, as sometimes misinterpreted. The highest VEGF protein levels were reported in placentas from preeclamptic women compared to normal pregnancies in Sahay’s study (43 PE pregnancies compared to 51 normal pregnancies) [23]. A significantly higher VEGF expression was obtained by Akercan in placenta samples of preeclamptic patients compared to that of controls [24].

HIF-1α showed a similar distribution. The control placentas were uniformly negative (score 0), while the IUGR group displayed either weak, focal positivity (score 1, 25%) or strong, diffuse expression (score 3, 75%) (*p* < 0.0001), consistent with hypoxia-induced upregulation.

IUGR pregnancies are associated with disruptions in miRNA, involving key mediators of angiogenesis. This includes a lower expression of HIF1A in the preeclamptic group, as observed by Song [19] and Ashraf [4].

In addition, Admati revealed massive dysregulation of gene expression, for example, of genes related to sFLT1/placental growth factor (PGF) [22]. In a study by Schröder-Heurich, increased placental HIF1 levels led to abnormal placentation through impaired uterine spiral artery remodeling and IUGR. Therapeutic targeting of the HIF pathway by injecting acriflavine improved placental development in his research, and also improved both maternal and fetal health [25]. Hypoxia-inducible factor 1 alpha (HIF-α) dysregulation contributes to alterations in matrix metalloproteinases (MMPs), cytokines, and endothelins (ETs) and aberrations in CD31+ cells and soluble HLA-G, resulting in endothelial dysfunction and reduced trophoblast invasion. This may play a role in predicting IUGR [26]. Several researchers have observed 429 differentially expressed genes that compromised the inflammatory response in the placenta of preeclamptic pregnancies [27].

In preterm preeclampsia, PlGF and VEGFR-1 displayed a differential abundance in both soluble and EV fractions, whereas angiogenin, CD40L, endoglin, galectin-1, and TIMP1 were changed only in the soluble fraction [28,29].

Until now, we have highlighted the importance of endothelial dysfunction in the pathophysiology of preeclampsia, but the mechanism of how it occurs remains uncertain. Researchers have pointed out the predictive value of serum-soluble vascular endothelial growth factor receptor 1 (VEGFR-1) in IUGR associated with PE [30]. Serum VEGFR-1 can be used as a biomarker in the prediction, diagnosis, and risk management of women with subtypes of PE [28].

The placenta is a site at which immunomodulatory actions occur, based on the communication between maternal immune cells and fetal trophoblasts [31,32,33]. An imbalance between the M1(CD68) and M2(CD163) macrophages in the placenta is observed during pregnancies with IUGR [34]. The immune tolerance of the maternal organism to the fetus is promoted by macrophages and NK cells. Macrophages may either behave as pro-inflammatory cells or help to resolve inflammation [15,35]. The PE-specific immune cell network is regulated by pro-inflammatory macrophages, which provide new ideas about the pathogenesis of PE [36].

The association between macrophages and placental disorders and defective angiogenesis was only found in PE with IUGR, and not in PE pregnancies without IUGR, according to Song. In his research, he observed a diminished value of CD68 and increased CD163 in PE, as a consequence of a shift in the balance of pro- and anti-inflammatory macrophages within the decidua basalis [34]. This may lead to the incorrect remodeling of the spiral arteries [15]. In our research, CD68 highlighted Hofbauer cells exclusively within the villous stroma, without involving the decidua, showing an increased density in IUGR placentas (scores 1 and 3). This is consistent with enhanced inflammatory/repair activity rather than “villitis” as Song suggested. The control placentas presented a physiological low-density macrophage pattern (score 0), whereas the IUGR placentas showed either mild Hofbauer cell infiltration (score 1) or extensive macrophage accumulation (score 3) (*p* < 0.0001). Our findings are consistent with another study, in which the intensity of CD68 was significant in the IUGR group compared to the control group (*p*  <  0.001). The mRNA level results for CD68 were also parallel to the immunostaining results [37]. This has also been observed by other researchers. More than 50% of the decidual macrophages were positive for the macrophage marker CD68 [15]. Fetal-origin Hofbauer and maternal-origin TREM2 macrophages were revealed as surprising main actors in early preeclampsia, while late preeclampsia showed minimal cellular impact on the placenta [22,38]. However, the role of macrophages in fetal growth restriction remains limited [38].

Endothelial progenitor cells (EPCs) are key regulators of wound healing and angiogenesis. During gestation, circulating EPCs, characterized by markers such as CD34, CD133, and VEGFR2, exhibit dynamic changes in their expression profiles. In the first trimester, their high expression indicates vasculogenic potential because of the increased demands of placental and fetal development [25]. They have been investigated in infants born after intrauterine growth restriction (IUGR), who are at risk of developing arterial hypertension at adulthood. Simoncini described the impaired functionality of EPCs in the cord blood of low-birth-weight newborns [39]. EPCs isolated from the bone marrow of IUGR infants displayed a decreased proportion of CD31+ versus CD146+ staining, and decreased CD34 expression through flow cytometry and immunofluorescence [39]. In our study, the CD34 expression remained strongly positive in endothelial cells in both groups, confirming a preserved villous vascular density, despite malperfusion compatible with a syncytiotrophoblast-driven, not endothelial-driven, hypoxic response.

Epithelial cells primarily employ cytokeratin in their cytoskeleton, while mesenchymal cells use vimentin. An exchange happens during the epithelial–mesenchymal transition (EMT). Cytokeratin-positive epithelial cells begin to express vimentin [40]. Immunofluorescence staining for cytokeratin 7 and vimentin for cellular and molecular biology was assessed to study the invasive properties of chorionic trophoblast cells. An increase in vimentin compared to cytokeratin 7 was observed in defective placentation, while the chorionic trophoblast cells failed to syncytialize and did not invade [41]. In another study, Hawkins observed the process of differentiation from proliferative cytotrophoblasts to the syncytiotrophoblast through β-hCG and cytokeratin-7 expression [42]. Impaired extravillous trophoblast invasion followed by incomplete spiral artery remodeling was investigated through CCR7 co-stained with cytokeratin 7 (CK7). The results showed that CCR7 and CK7 are involved in PE [43]. According to our research, CK7 staining was uniformly strong and continuous in both groups (score 3), demonstrating preserved trophoblastic integrity without evidence of impaired syncytialization.

All these research efforts could lead to a potential new avenue for treating this pregnancy disorder by finding biomarkers that can be used as novel therapeutic targets in PE patients to improve fetal growth [4]. Research on in utero gene therapy has advanced, but ethical and safety concerns persist [44,45]. However, neither the ISUOG nor the SMFM has included placental histopathological findings in their IUGR definition. In addition, placental insufficiency does not reflect neonatal complications, a fact also observed in our study [46]. In our study, histopathological evaluation of H&E-stained sections was conducted in accordance with the Amsterdam Placental Workshop Group Consensus Statement, which emphasizes a descriptive and pattern-based approach to placental lesions rather than a strictly quantitative scoring system. At present, there is no universally accepted or validated quantitative scoring method for routine H&E assessment of placental maternal vascular malperfusion features. In this context, we chose to avoid applying non-validated or arbitrary numerical scores to H&E morphology. By contrast, immunohistochemical analyses were evaluated using a standardized semi-quantitative 0–3 scoring system, as such grading systems are well established and widely accepted for immunohistochemical evaluation. Although the full scoring scale was applied during assessment, staining patterns in our cohort were observed predominantly at the extremes of the scale, and no intermediate (score 2) intensity was identified.

The main limitation of the work is that it is a single-center study on a small sample size, which inevitably reduces statistical power and limits the generalizability of the findings. There is an increased risk of type I and type II statistical errors. Also, we only validated the results in clinical trials, without in vitro or animal experiments. Further studies are mandatory.

## 5. Conclusions

Overall, the clinical and histopathological findings demonstrate clear distinctions between pregnancies complicated by IUGR and healthy controls. Marked differences were observed in antenatal monitoring parameters based on ultrasound and Doppler evaluations.

A histopathological assessment using H&E staining, together with immunohistochemistry, confirmed a strong association between IUGR and placental ischemia, characterized by villous maldevelopment and features of maternal vascular malperfusion. Significant differences were identified in the expression of VEGF, HIF-1α, and CD68, reflecting hypoxic and inflammatory responses, while CD34 and CK7 remained unchanged across groups, indicating maintained villous endothelial integrity and trophoblastic continuity.

These findings underline the diagnostic value of placental examination in IUGR and support its role in correlating structural lesions with antenatal Doppler abnormalities. However, it is important to add that this is a pilot study without definitive evidence. Further studies are warranted to clarify the biological pathways involved and their potential relevance for future clinical applications.

## Figures and Tables

**Table 1:** Demographic characteristics and ultrasound measurements.

Variables		Groups			*p*-Value *
		IUGR (16) No. (%)	Control (18) No. (%)	Total (34) No. (%)	
Para	1	10 (62.5)	13 (72.2)	23 (67.6)	0.75
	2	3 (18.8)	2 (11.1)	5 (14.7)	
	3	2 (12.5)	3 (16.7)	5 (14.7)	
	4	1 (6.2)	0 (0.0)	1 (2.9)	
Gestation	1	6 (37.5)	8 (44.4)	14 (41.2)	0.97
	2	5 (31.2)	5 (27.8)	10 (29.4)	
	3	2 (12.5)	3 (16.7)	5 (14.7)	
	4	2 (12.5)	1 (5.6)	3 (8.8)	
	6	1 (6.2)	1 (5.6)	2 (5.9)	
Smoking	Yes	6 (37.5)	4 (22.2)	10 (29.4)	0.32
Alcohol	No	16 (100)	18 (100)	34 (100)	
Drugs	No	16 (100)	18 (100)	34 (100)	
Pathologies	DZ II	2 (12.5)	0 (0)	2 (5.9)	0.007
	Gestational diabetes	1 (6.2)	0 (0)	1 (2.9)	
	Hypothyroidism	1 (6.2)	0 (0)	1 (2.9)	
	HTA	1 (6.2)	0 (0)	2 (2.9)	
	No	10 (62.5)	18 (100)	28 (82.4)	
	Thrombophilia	1 (6.2)	0 (0)	1 (2.9)	
Fetal gender	F	9 (56.2)	6 (33.3)	15 (44.1)	0.17
	M	7 (43.8)	12 (66.7)	19 (55.9)	
APGAR	4	1 (6.2)	0 (0)	1 (2.9)	0.14
	5	0 (0)	1 (5.6)	1 (2.9)	
	6	1 (6.2)	0 (0)	1 (2.9)	
	7	1 (6.2)	0 (0)	1 (2.9)	
	8	7 (43.8)	5 (27.8)	12 (35.3)	
	9	6 (37.5)	12 (66.7)	18 (52.9)	
NICU	No	14 (87.5)	18 (100)	32 (94.1)	0.12
	Yes	2 (12.5)	0 (0)	2 (5.9)	
Environment	U	13 (81.2)	12 (66.7)	25 (73.5)	0.33
Education	College	3 (18.8)	1 (5.6)	4 (11.8)	0.62
	Elementary school	2 (12.5)	3 (16.7)	5 (14.7)	
	High school	5 (31.2)	9 (50)	14 (41.2)	
	University	6 (37.5)	5 (27.8)	11 (32.4)	
CTG	Normal	2 (12.5)	17 (94.4)	19 (55.9)	0.0001
	<10th percentile	14 (87.5)	1 (5.6)	15 (44.1)	
Ultrasound measurements	<10th percentile	16 (100)	1 (5.6)	17 (50)	0.0001
	Normal	0 (0)	17 (94.4)	17 (50)	
PI uterine artery in first trimester	PI < 1.69	0 (0)	17 (94.4)	17 (50)	0.0001
	PI > 1.69	16 (100)	1 (5.6)	17 (50)	
Uterine Doppler at 20–22 weeks	<95th percentile	0 (0)	16 (88.9)	16 (47.1)	0.0001
	>95th percentile	16 (100)	2 (11.1)	18 (52.9)	
Notch	No	8 (50)	18 (100)	26 (76.5)	0.001
	Yes	8 (50)	0 (0)	8 (23.5)	

IUGR—intrauterine growth restriction; CTG—cardiotocography; NICU—neonatal intensive care unit; PI—pulsatility index; *—we used a chi-squared test.

**Table 2:** Placental findings.

Variables		Lots		*p*-Value
	IUGR (*n* = 16)	Control (*n* = 18)	Total (*n* = 34)	
H&E (hematoxylin–eosin)				
Mild villous changes: minimal stromal edema and mild vascular congestion; preserved architecture	4 (25%)	0 (0%)	4 (11.8%)	0.0001
Normal villous structure; no significant abnormalities	0 (0%)	17 (94.4%)	17 (50.0%)	
Severe villous abnormalities: caliber reduction, stromal fibrosis, and vascular changes consistent with advanced maternal vascular malperfusion	12 (75%)	1 (5.6%)	13 (38.2%)	
Fibrin deposits and intervillous thrombi				
Accentuated perivillous fibrin with occasional intervillous thrombi	12 (75%)	1 (5.6%)	13 (38.2%)	0.0001
Minimal fibrin deposition (physiological limits)	0 (0%)	17 (94.4%)	17 (50.0%)	
Moderate fibrin with occasional intervillous thrombi	3 (18.8%)	0 (0%)	3 (8.8%)	
Moderate fibrin with rare intervillous thrombi	1 (6.2%)	0 (0%)	1 (2.9%)	
Syncytial knots				
Marked increase	12 (75%)	1 (5.6%)	13 (38.2%)	0.0001
Moderate increase	4 (25%)	0 (0%)	4 (11.8%)	
Physiological number	0 (0%)	17 (94.4%)	17 (50.0%)	
Anti-VEGF				
Score 1—weak physiological staining	0 (0%)	17 (94.4%)	17 (50.0%)	0.0001
Score 3—strong, diffuse cytoplasmic expression	16 (100%)	1 (5.6%)	17 (50.0%)	
Anti-HIF-1α				
Score 0—negative	0 (0%)	17 (94.4%)	17 (50.0%)	0.0001
Score 1—weak, focal expression	4 (25%)	0 (0%)	4 (11.8%)	
Score 3—strong, diffuse expression	12 (75%)	1 (5.6%)	13 (38.2%)	
Anti-CD34				
Score 3—strong endothelial staining, preserved microvascular density	14 (87.5%)	18 (100%)	32 (94.1%)	0.12
Score 3—strong endothelial staining in less vascular areas (variant distribution)	2 (12.5%)	0 (0%)	2 (5.9%)	
Anti-CK7				
Score 3—strong, continuous trophoblast staining	16 (100%)	18 (100%)	34 (100%)	—
Anti-CD68				
Score 0—physiological Hofbauer cell density	0 (0%)	17 (94.4%)	17 (50.0%)	0.0001
Score 1—few scattered Hofbauer cells	4 (25%)	0 (0%)	4 (11.8%)	
Score 3—numerous Hofbauer cells, increased inflammatory/repair activity	12 (75%)	1 (5.6%)	13 (38.2%)	

VEGF—vascular endothelial growth factor; HIF-1α—hypoxia-inducible factor 1 alpha; CK7—cytokeratin 7; CD34—endothelial cell marker indicating villous microvascular density; CD68—Hofbauer cell (villous macrophage) marker; H&E—hematoxylin–eosin; IUGR—intrauterine growth restriction; PI—pulsatility index. Scores (0–3)—semi-quantitative immunohistochemical staining intensity scale: 0 = absent; 1 = weak/focal; 2 = moderate (not observed in this cohort); 3 = strong/diffuse. *p*-values were calculated using a chi-squared test.

**Table 3:** Bivariate analysis of anthropometric indicators.

	Groups	*n*	Mean	Std. Deviation	*p*-Value *
Age	IUGR	16	31.25	5.247	0.33
	Control	18	29.28	6.388	
Weight	IUGR	16	89.06	13.284	0.022
	Control	18	79.89	8.724	
BMI	IUGR	16	29.56	4.320	0.008
	Control	18	25.89	3.160	
Gestation at delivery	IUGR	16	37.19	1.223	0.0001
	Control	18	39.11	1.183	
Fetal birth weight	IUGR	16	2115.63	252.42	0.0001
	Control	18	3222.35	520.93	
IR MCA	IUGR	16	0.655	0491	0.0001
	Control	18	0.869	0776	
Placental weight	IUGR	16	328.75	41.773	0.0001
	Control	18	576.67	97.317	
Weight gain	IUGR	16	17.13	3.519	0.0001
	Control	18	12.56	2.593	

BMI—body mass index; IUGR—intrauterine growth restriction; IR MCA—resistivity index in the middle cerebral artery. Student’s *t*-test: data were expressed as mean ±SD.

## Data Availability

The data presented in this study are available from the corresponding author and the coordinator of this study upon request.

## Abbreviations

The following abbreviations are used in this manuscript:

IUGR	intrauterine growth restriction
ISUOG	International Society of Ultrasound in Obstetrics and Gynecology
SMFM	Society for Maternal–Fetal Medicine
ACOG	American College of Obstetricians and Gynecologists
EFW	estimated fetal weight
SGA	small for gestational age
PE	preeclampsia
VEGF	vascular endothelial growth factor
sFlt-1	soluble fms-like tyrosine kinase 1 (anti-angiogenic factor)
HIF-1α	hypoxia-inducible factor 1 alpha
CK7	cytokeratin 7
BMI	body mass index
NICU	neonatal intensive care unit
PI	pulsatility index
RI	resistivity index
MCA	middle cerebral artery
H&E	hematoxylin and eosin
IHC	immunohistochemistry
BW	birth weight
EPCs	endothelial progenitor cells
EMT	epithelial–mesenchymal transition
PlGF	placental growth factor
MVM	maternal vascular malperfusion

## References

[1] Mansour Ghanaei M., Amir Afzali S., Morady A., Mansour Ghanaie R., Asghari Ghalebin S.M., Rafiei E., Kabodmehri R. (2022). Intrauterine growth restriction with and without pre-eclampsia: Pregnancy outcome and placental findings. J. Obstet. Gynecol. Cancer Res..

[2] Lees C.C., Stampalija T., Baschat A., da Silva Costa F., Ferrazzi E., Figueras F., Hecher K., Kingdom J., Poon L.C., Salomon L.J. (2020). ISUOG Practice Guidelines: Diagnosis and management of small-for-gestational-age fetus and fetal growth restriction. Ultrasound Obstet. Gynecol..

[3] Salomon L.J., Alfirevic Z., Da Silva Costa F., Deter R.L., Figueras F., Ghi T.A., Glanc P., Khalil A., Lee W., Napolitano R. (2019). ISUOG Practice Guidelines: Ultrasound assessment of fetal biometry and growth. Ultrasound Obstet. Gynecol..

[4] Ashraf U.M., Hall D.L., Rawls A.Z., Alexander B.T. (2021). Epigenetic processes during preeclampsia and effects on fetal development and chronic health. Clin. Sci..

[5] Docheva N., Arenas G., Nieman K.M., Lopes-Perdigao J., Yeo K.T.J., Rana S. (2022). Angiogenic biomarkers for risk stratification in women with preeclampsia. Clin. Chem..

[6] Melchiorre K., Giorgione V., Thilaganathan B. (2022). The placenta and preeclampsia: Villain or victim?. Am. J. Obstet. Gynecol..

[7] Liu J., Zhao M., Zhang S., Shi Y. (2025). HIF-1α-mediated inhibition of the sFlt-1/sENG/TNF-α pathway promotes angiogenesis to ameliorate pre-eclampsia. J. Mol. Histol..

[8] Bakrania B.A., Spradley F.T., Drummond H.A., LaMarca B., Ryan M.J., Granger J.P. (2021). Preeclampsia: Linking placental ischemia with maternal endothelial and vascular dysfunction. Compr. Physiol..

[9] Roeckner J.T., Pressman K., Odibo L., Duncan J.R., Odibo A.O. (2021). Outcome-based comparison of SMFM and ISUOG definitions of fetal growth restriction. Ultrasound Obstet. Gynecol..

[10] Restriction F.G. (2021). ACOG Practice Bulletin. Obstet. Gynecol..

[11] Danciu B.M., Simionescu A.A. (2025). Optimizing fetal surveillance in fetal growth restriction: A narrative review of the role of the computerized cardiotocographic assessment. J. Clin. Med..

[12] Palei A.C., Granger J.P., Spradley F.T. (2021). Placental ischemia says “NO” to proper NOS-mediated control of vascular tone and blood pressure in preeclampsia. Int. J. Mol. Sci..

[13] Fruci S., Salvi S., Moresi S., Gallini F., Dell’Aquila M., Arena V., Di Stasio E., Ferrazzani S., De Carolis S., Lanzone A. (2023). Pravastatin for severe preeclampsia with growth restriction: Placental findings and infant follow-up. Eur. J. Obstet. Gynecol. Reprod. Biol..

[14] Khong T.Y., Mooney E.E., Ariel I., Balmus N.C.M., Boyd T.K., Brundler M.-A., Derricott H., Evans M.J., Faye-Petersen O.M., Gillan J.E. (2016). Sampling and definitions of placental lesions. Amsterdam placental workshop group consensus statement. Arch. Pathol. Lab. Med..

[15] Vishnyakova P., Poltavets A., Nikitina M., Midiber K., Mikhaleva L., Muminova K., Potapova A., Khodzhaeva Z., Pyregov A., Elchaninov A. (2021). Expression of estrogen receptor α by decidual macrophages in preeclampsia. Biomedicines.

[16] Bujorescu D.L., Raţiu A.C., Motoc A.G.M., Cîtu I.C., Sas I., Gorun I.F., Gorun O.-M., Folescu R., Gurguş D. (2023). Placental pathology in early-onset fetal growth restriction: Insights into fetal growth restriction mechanisms. Rom. J. Morphol. Embryol..

[17] Stampalija T., Lees C., Ghi T., Cornette J., Gyselaers W., Ferrazzi E., Mousa T., Spaanderman M., Thilaganathan B., Valensise H. (2025). ISUOG Consensus Statement on maternal hemodynamic assessment in hypertensive disorders of pregnancy and fetal growth restriction. Ultrasound Obstet. Gynecol..

[18] Schreiber V., Hurst C., da Silva Costa F., Stoke R., Turner J., Kumar S. (2023). Definitions matter: Detection rates and perinatal outcome for infants classified prenatally as having late fetal growth restriction using SMFM biometric vs ISUOG/Delphi consensus criteria. Ultrasound Obstet. Gynecol..

[19] Song W., Guo Q., Puttabyatappa M., Elangovan V.R., Wang J., Li F., Liu F., Bi X., Li H., Fu G. (2024). FGR-associated placental insufficiency and capillary angiogenesis involves disruptions in human placental miRNAs and mRNAs. Heliyon.

[20] Chiorean D.M., Cobankent Aytekin E., Mitranovici M.I., Turdean S.G., Moharer M.S., Cotoi O.S., Toru H.S. (2024). Human placenta and evolving insights into pathological changes of preeclampsia: A comprehensive review of the last decade. Fetal Pediatr. Pathol..

[21] Albogami S.M., Al-Kuraishy H.M., Al-Maiahy T.J., Al-Buhadily A.K., Al-Gareeb A.I., Alorabi M., Alotaibi S.S., De Waard M., Sabatier J.-M., Saad H.M. (2022). Hypoxia-inducible factor 1 and preeclampsia: A new perspective. Curr. Hypertens. Rep..

[22] Admati I., Skarbianskis N., Hochgerner H., Ophir O., Weiner Z., Yagel S., Solt I., Zeisel A. (2023). Two distinct molecular faces of preeclampsia revealed by single-cell transcriptomics. Med.

[23] Sahay A.S., Jadhav A.T., Sundrani D.P., Wagh G.N., Mehendale S.S., Chavan-Gautam P., Joshi S.R. (2018). VEGF and VEGFR1 levels in different regions of the normal and preeclampsia placentae. Mol. Cell. Biochem..

[24] Akercan F., Cirpan T., Terek M.C., Ozcakir H.T., Giray G., Sagol S., Karadadas N. (2008). The immunohistochemical evaluation of VEGF in placenta biopsies of pregnancies complicated by preeclampsia. Arch. Gynecol. Obstet..

[25] Schröder-Heurich B., Beckmann J., von Versen-Höynck F. (2025). Endothelial progenitor cells in Life, Pregnancy and Disease. Expert Rev. Mol. Med..

[26] Chiang Y.T., Seow K.M., Chen K.H. (2024). The pathophysiological, genetic, and hormonal changes in preeclampsia: A systematic review of the molecular mechanisms. Int. J. Mol. Sci..

[27] Chou A., Hiatt O., Davidson B., Reynolds P.R., Pickett B.E., Arroyo J.A. (2025). Vaping in Pregnancy: Unraveling Molecular Drivers of Preeclampsia and Fetal Growth Restriction. Int. J. Mol. Sci..

[28] Than N.G., Romero R., Fitzgerald W., Gudicha D.W., Gomez-Lopez N., Posta M., Zhou F., Bhatti G., Meyyazhagan A., Awonuga A.O. (2024). Proteomic profiles of maternal plasma extracellular vesicles for prediction of preeclampsia. Am. J. Reprod. Immunol..

[29] Mitranovici M.I., Chiorean D.M., Moraru R., Moraru L., Caravia L., Tiron A.T., Craina M., Cotoi O.S. (2024). Understanding the pathophysiology of preeclampsia: Exploring the role of antiphospholipid antibodies and future directions. J. Clin. Med..

[30] Şalk S., Yurtcu N., Çetin A. (2022). Predictive and diagnostic value of serum sVEGFR-1 level in women with preeclampsia: A prospective controlled study. Turk. J. Obstet. Gynecol..

[31] Smith J., Powell M., Cromartie W., Smith S., Jones K., Castillo A., Shaw J., Editone J., Howard A., Tatum R. (2024). Intrauterine growth-restricted pregnant rats, from placental ischemic dams, display preeclamptic-like symptoms: A new rat model of preeclampsia. Physiol. Rep..

[32] Ortega M.A., Fraile-Martinez O., Garcia-Montero C., Saez M.A., Álvarez-Mon M.A., Torres-Carranza D., Álvarez-Mon M., Bujan J., García-Honduvilla N., Bravo C. (2022). The pivotal role of the placenta in normal and pathological pregnancies: A focus on preeclampsia, fetal growth restriction, and maternal chronic venous disease. Cells.

[33] Hebeda C.B., Savioli A.C., Scharf P., de Paula-Silva M., Gil C.D., Farsky S.H.P., Sandri S. (2022). Neutrophil depletion in the pre-implantation phase impairs pregnancy index, placenta and fetus development. Front. Immunol..

[34] Song W., Wang F., Li X., Liu F., Yu T., Fan X., Li M., Guo Q. (2024). The discrepancy distribution of macrophage subsets in preeclampsia placenta with or without fetal growth restriction from a small cohort. Ginekol. Pol..

[35] Pang B., Hu C., Li H., Nie X., Wang K., Zhou C., Yi H. (2023). Myeloidderived suppressor cells: Escorts at the maternal–fetal interface. Front. Immunol..

[36] Fei H., Lu X., Shi Z., Liu X., Yang C., Zhu X., Lin Y., Jiang Z., Wang J., Huang D. (2025). Deciphering the preeclampsia-specific immune microenvironment and the role of pro-inflammatory macrophages at the maternal–fetal interface. eLife.

[37] Calis P., Gundogdu A.C., Turgut E., Seymen C.M., Saglam A.S., Karcaaltincaba D., Kaplanoglu G.T. (2024). Do small for gestational age fetuses have placental pathologies?. Arch. Gynecol. Obstet..

[38] Bezemer R.E., Faas M.M., Van Goor H., Gordijn S.J., Prins J.R. (2024). Decidual macrophages and Hofbauer cells in fetal growth restriction. Front. Immunol..

[39] Simoncini S., Coppola H., Rocca A., Bachmann I., Guillot E., Zippo L., Dignat-George F., Sabatier F., Bedel R., Wilson A. (2021). Endothelial colony-forming cells dysfunctions are associated with arterial hypertension in a rat model of intrauterine growth restriction. Int. J. Mol. Sci..

[40] Kuburich N.A., den Hollander P., Pietz J.T., Mani S.A. (2022). Vimentin and cytokeratin: Good alone, bad together. Seminars in Cancer Biology.

[41] Choudhury J., Richardson L.S., Urrabaz-Garza R., Jacob J., Kammala A.K., Menon R. (2025). Chorionic trophoblast cells demonstrate functionally different phenotypes from placental trophoblasts. Biol. Reprod..

[42] Hawkins A., Pantazi P., Yang L., Coyne C.B., Bokun V., Lemme-Dumit J.M., Pasetti M.F., Barnett S., Culley F.J., Holder B. (2024). Long-term culture and passaging of term trophoblast for the investigation of syncytiotrophoblast function. Placenta.

[43] Liu Z., He J., Jin P., Ran Y., Yin N., Qi H. (2023). CCL21/CCR7 axis contributes to trophoblastic cell migration and invasion in preeclampsia by affecting the epithelial mesenchymal transition via the ERK1/2 signaling pathway. Biology.

[44] Majumder S., Moriarty K.L., Lee Y., Crombleholme T.M. (2024). Placental Gene Therapy for Fetal Growth Restriction and Preeclampsia: Preclinical Studies and Prospects for Clinical Application. J. Clin. Med..

[45] Song W., Guo Q., Puttabyatappa M., Elangovan V.R., Wang J., Li F., Liu F., Bi X., Li H., Fu G. (2022). Sex-Specific Disruption in Human Placental miRNAs and mRNAs Involved in IUGR Placental Insufficiency and Capillary Angiogenesis. Res. Sq..

[46] Rodriguez-Sibaja M.J., Lopez-Diaz A.J., Valdespino-Vazquez M.Y., Acevedo-Gallegos S., Amaya-Guel Y., Camarena-Cabrera D.M., Lumbreras-Marquez M.I. (2024). Placental pathology lesions: International Society for Ultrasound in Obstetrics and Gynecology vs Society for Maternal-Fetal Medicine fetal growth restriction definitions. Am. J. Obstet. Gynecol. MFM.

